# Neurodevelopmental and social determinants of school support received by children born preterm

**DOI:** 10.1038/s41390-025-04287-4

**Published:** 2025-08-04

**Authors:** Anna-Veera Seppänen, Véronique Pierrat, Laetitia Marchand-Martin, Marie-Laure Charkaluk, Jean-Baptiste Muller, Stéphane Marret, Jennifer Zeitlin, Mariane Sentenac

**Affiliations:** 1 Université Paris Cité, INSERM UMR 1153, Centre for Research in Epidemiology and Statistics (CRESS), Obstetric, Perinatal, Paediatric Life Course Epidemiology Research Team (OPPaLE), Paris, France; 2 Réanimation et Médecine Néonatale, Centre Hospitalier Intercommunal de Créteil, Créteil, France; 3 Department of Neonatology, Saint Vincent de Paul Hospital, GHICL, Lille, France; 4 La Faculté de Médecine, Maïeutique, Sciences de la Santé, Lille Catholic University, Lille, France; 5 Department of Neonatal Medicine, Intensive Care, and Neuropediatrics, Rouen University Hospital, Normandy University, Rouen, France; 6 INSERM U1254, Neovascular Team, Perinatal Handicap, Institute of Biomedical Research and Innovation, Normandy University, Rouen, France

## Abstract

**Background:**

Children born preterm face higher risks of neurodevelopmental difficulties that, with social vulnerabilities, may impair school performance. We described and assessed determinants of receiving school support in preterm-born children in France.

**Methods:**

We used data from the prospective population-based cohort of births before 35 weeks’ gestation in France, EPIPAGE-2, to estimate crude rates and adjusted relative risks (using multivariable, mixed-effects generalized linear models) of receiving school support at age five, by children’s neurodevelopment at five, sociodemographic characteristics, and region.

**Results:**

Out of 3,007 children, 99% attended mainstream school at age five, of whom 9% received school support. Support was more often received by boys (11%; aRR = 1.37) than girls (6%), children born at 24–27 weeks’ gestation (21%; aRR = 2.78 compared to 32–34 weeks), and children with moderate or severe neurodevelopmental impairments (MSNDI: cerebral palsy, cognitive impairment, visual impairment or blindness, and/or hearing impairment or deafness) (39%; aRR = 17.25 compared to none). Receiving support was not associated with sociodemographic characteristics, after adjusting for covariates.

**Conclusions:**

Neurodevelopmental impairment is a key determinant for receiving school support. However, 9% of the cohort and under 40% of children with MSNDI were receiving support, raising questions on whether unmet needs for school support exist in France.

**Impact:**

- This study provides an overview of school support received at age five by children born before 35 weeks’ gestation in France, and associated determinants
- Less than 10% of the total cohort and 40% of children with a moderate or severe neurodevelopmental impairment were receiving school support
- Cognitive and neurodevelopmental impairments were key determinants for receiving school support, but sociodemographic characteristics were not
- Our results raise questions about whether unmet needs for school support exist, calling for further research on the support available in schools, decision-making processes for allocating them, and the psychosocial and academic consequences of their provision on children.

## Introduction

Preterm births (PTB) before 35 weeks of gestational age (GA) represent approximately 3% of all births in France.^1^ PTB is associated with an increased risk of neurodevelopmental difficulties, including cerebral palsy (CP) as well as motor, hearing, vision, cognitive and behavioural impairment.^2–6^ The consequences of PTB can lead to learning difficulties, impact school readiness,^7,8^ and influence school performance and academic skills.^9–11^ Research shows that children born preterm have a greater need for special education, school support and assistance compared to children born at term.^9,10,12,13^ The need for special education increases with decreasing GA,^12^ and school assistance is also used by children with minor and moderate neurodevelopmental difficulties.^5^ Furthermore, the child’s language, cognitive^14,15^ and motor development, behaviour,^16–18^ and school performance^7,19^ can be influenced by family socioeconomic and demographic vulnerabilities. As the risk of having preterm delivery is higher in women with socioeconomic hardship,^20^ social vulnerabilities may create a double burden of prematurity and risk factors for poor cognitive development, that may impact future academic opportunities.^21^

Early childhood education helps children to develop their academic and socioemotional skills,^22^ prepares them for primary school, and plays an important role in the early detection of educational support needs.^23^ In 1994, UNESCO urged all countries to prioritize inclusive education, to ensure equal opportunities for all children to learn within mainstream schools, regardless of developmental or social vulnerabilities,^23^ and the 2030 Agenda for Sustainable Development calls for inclusive and equitable early childhood education, in order to *leave no one behind*.^24^ Addressing the additional needs of children with social vulnerabilities may be necessary to ensure access to inclusive education and narrow the future gap in opportunities across the social gradient.^25^ High-quality early childhood education has been found particularly beneficial for children from disadvantaged backgrounds,^26^ highlighting the importance to assess the equitability of learning opportunities.

In France, the official age of pre-school enrolment is 3 years’ chronological age. Preschool is free of charge from 3 years of age until entry to primary school at age six.^27^ For children needing support for specific illnesses, a Personalized Accommodation Plan^28^ is established, defining whether the child needs adapted meals (e.g., in case of allergies) or physical activities, or help with taking medications (e.g., for treating asthma), for instance. If more support is needed, a Personalized Schooling Plan^28^ for children with disabilities or chronic illness is developed at the Departmental Medical Home for Persons with Disabilities—a local administrative office^28^—to help organise the most appropriate schooling for the child. The plan defines whether the child should attend an inclusive or special education classroom, and the types of support needed, including a teacher or psychologist from a special needs support network, therapeutic and rehabilitation activities, a school support assistant, and technical or material aid (for instance, adapted seat or computer).^28–30^

Recent research in a European sample of very preterm-born ( < 32 weeks of GA) 5-year-olds reported that 23% were receiving school support, but rates varied markedly across countries.^31^ School support was consistently higher in children with risk factors for neurodevelopmental difficulties (such as perinatal morbidities).^31^ However, the European study did not include moderately PTB, and was not able to classify children by neurodevelopmental disability, with limited ability to assess the extent to which services were targeted to children needing support.

The aim of this study was to describe the types of school support received by preschool-aged (five-year-old) children born before 35 weeks’ GA in France, and to assess whether variations in school support exists by children’s neurodevelopment, family sociodemographic characteristics and region of residence.

## Methods

### Study population and data collection

We used data collected in the French prospective population-based cohort of children born preterm, *Etude Épidémiologique sur les Petits Âges Gestationnels* – EPIPAGE-2.^32^ The cohort included all infants born between 22–26 weeks’ GA over a period of eight months, 27–31 weeks’ GA over six months, and 32–34 weeks’ GA over a period of five weeks, from March 2011 onwards, in all maternity units in 25 (all but one) French regions.^32^ A reference sample born at 37–40 weeks’ GA was obtained from a sub-sample of 592 children who are part of the contemporary ELFE (Étude Longitudinale Française depuis l’Enfance) cohort, using the EPIPAGE-2 protocol.^33^ Family sociodemographic data at the time of birth were collected in maternity units via interviews with mothers, and perinatal and birth-related data were extracted from obstetric and neonatal records. At the 5-year follow-up, tests were performed by psychologists to assess the child’s cognitive impairment, using the Wechsler Preschool and Primary Scale of Intelligence (WPPSI, 4th edition), and by paediatricians, assessing CP, using diagnostic criteria of the Surveillance of CP in Europe network and graded using the Gross Motor Function Classification System (GMFCS); developmental coordination disorders, using the Movement Assessment Battery for Children 2nd edition (M-ABC2); and hearing and vision impairment, based on parent report and medical records. Parental consent was obtained to collect information on the severity of disability from rehabilitation centres for children whose disability prevented them from participating in these clinical exams. Parents also filled in a questionnaire about their family and socioeconomic characteristics, the child’s school participation and school support, and behavioural difficulties reported with the Strengths and Difficulties Questionnaire (SDQ).

### Data on perinatal and birth-related characteristics

Data on birth-related characteristics included: child’s sex; GA in completed weeks; birthweight ( ≥ 10^th^ centile 3^rd^–9^th^ centile or <3^rd^ centile) based on curves adapted to the French population^34^; multiple pregnancy (singleton vs multiples); parity (primiparous or multiparous); and presence of any severe neonatal morbidity (severe intraventricular haemorrhage [grades III-IV] or cystic periventricular leukomalacia based on ultrasound diagnosis, severe necrotising enterocolitis [Bell’s stages 2–3], severe retinopathy of prematurity [stage >3], severe bronchopulmonary dysplasia [oxygen for at least 28 days and persistent need for oxygen or ventilatory support at 36 weeks’ postmenstrual age], and/or late onset sepsis [positive blood culture after 72 h of life, associated with antibiotic administration for five days or more]).

### Data on schooling and school support

School participation data collected at the 5-year follow-up included: type of school attended (mainstream school, home school, specialized institution or not schooled); age at preschool entry (3 years of chronological age or younger, or delayed entry at 4 years of chronological age or older), level of schooling (delayed, usual, or in advance for age) and participation time (full or part time). Data also included whether or not the child had a “Personalized Accommodation Plan” or a “Personalized Schooling Plan” for children with disabilities or chronic illnesses, or any type of school support. School support was defined as any services or materials provided to the child at school, to help them attend, participate in activities and/or with learning, including: having a school support assistant; attending an inclusive classroom or special education classroom; receiving other professional aid than school support assistant (e.g., extra learning support or support teacher); having technical aid (e.g., adapted seat or table, inclined reading support, adapted computer, wheelchair or hearing aid); and/or having other types of aid (unspecified). Information on additional types of school support was retrieved from free text answers, including teaching unit for children with autism, and extra support from teacher or psychologist from a special needs assistance network specialising in learning difficulties. A composite variable “school support” was created, based on whether or not the child was receiving any of these types of school support at 5 years of age (yes/no). Having a “Personalized Accommodation Plan” or a “Personalized Schooling Plan” at the moment of data collection, in the absence of the other forms of support described above, was not considered equivalent to receiving school support, as significant delays can occur between establishing a plan and the actual provision of school support. See Supplementary Table S1 for the questions on school support in French and in English.

### Data on health and neurodevelopment at five years of age

The child’s health and neurodevelopment at 5 years of age were determined based on data from the medical exams and parent-report questionnaires, using definitions previously used in this cohort.^5^ The level of cognitive impairment was defined as none (an FSIQ score higher than 1 standard deviation (SD) below the reference mean), mild (an FSIQ score between 1 to 2 SD below the reference mean), and moderate or severe (an FSIQ score lower than 2 SD below the reference mean), using the FSIQ score distribution of the reference sample born at 37–40 weeks’ GA.^5^ CP was defined as none, mild (GMFCS level 1), or moderate or severe (GMFCS levels 2–5). Developmental coordination disorder was defined as an M-ABC2 score ≤5th centile based on the reference sample born at 37–40 weeks’ GA. Hearing impairment was classified as none, mild (hearing loss ≤40 dB), and moderate or severe (deafness or unilateral or bilateral hearing loss >40 dB, not corrected, or partially corrected with hearing aid). Vision impairment was classified as none (binocular visual acuity ≥5/10 or vision reported as normal in eye consultation within the past 12 months), mild (binocular visual acuity of 3.2–4.9/10), and moderate or severe (bilateral binocular visual acuity <3.2/10 or <1/10 (blindness)), measured with the Sander-Zanlonghi scale^35^ (with glasses if needed) or according to parent-report. Behavioural difficulties were defined as having an SDQ score ≥90th centile, based on the reference sample.

A composite variable reflecting the child’s level of neurodevelopmental impairment (NDI) was created according previously used methodology,^5^ defined as none; mild: mild CP, visual impairment, hearing impairment, or cognitive impairment, or any behavioural difficulties and/or developmental coordination disorder, but no moderate or severe impairment; and moderate/severe (MSNDI): moderate or severe CP, visual impairment, hearing impairment and/or cognitive impairment.

### Sociodemographic characteristics

Sociodemographic information included: mother’s age at delivery, mother’s educational level at the five-year follow-up (low: upper secondary or lower; intermediate: post-secondary, non-tertiary or short cycle tertiary; or higher: bachelor degree or equivalent or higher), child’s family situation (lives with single parent versus two parents), mother’s country of birth (France, or outside France), and region of residence at 5 years. Children who had emigrated and were residing outside of France at 5 years were excluded due to differences in schooling systems (*n* = 11).

### Statistical analyses

First, we described the main characteristics of the study sample, and compared them to non-participants using Chi² tests and Fisher’s exact test. Then, we described school type at 5 years of age, and school participation and school support received by children in mainstream school, by GA group and level of NDI. We compared crude rates of school support by child and birth-related characteristics, child’s neurodevelopment at 5 years, family sociodemographic characteristics, and by region, in univariable analyses using Chi² tests and Fisher’s exact test. We then described the crude proportions of children with school support by perinatal and family characteristics, stratified by level of NDI and cognitive impairment. Finally, we estimated the relative risk (RR) of receiving school support for variables that were associated with school support with a *p*-value of <0.01 in the univariable analysis, or selected based on the available literature. Severe neonatal morbidity was not included in these models as it may be on the pathway between GA, birth weight (BW) and school support, via NDI. We used multivariable, mixed-effects generalized linear models with a log link, Poisson distribution and a Huber-White robust variance estimator, accounting for intra-cluster correlation for siblings, with random intercepts for mothers, and adjusting for region. Using these models, we estimated adjusted RR (aRR) of school support for each region compared to the balanced grand mean, taking into account regional differences in case-mix, using the post-estimation command *contrast* in STATA 16.1 (StataCorp LLC, College Station, TX). Because of differences in the recruitment periods for different gestational ages, sampling weights were used for all analyses.

### Study ethics

EPIPAGE-2 was approved by the National Data Protection Authority (CNIL, DR-2016-290), the Consultative Committee on the Treatment of Data on Personal Health for Research Purposes (No. 16.263), and the Committee for the Protection of People Participating in Biomedical Research (2016-A00333-48). All participating families have provided written informed consent.

## Results

**Fig. 1:**
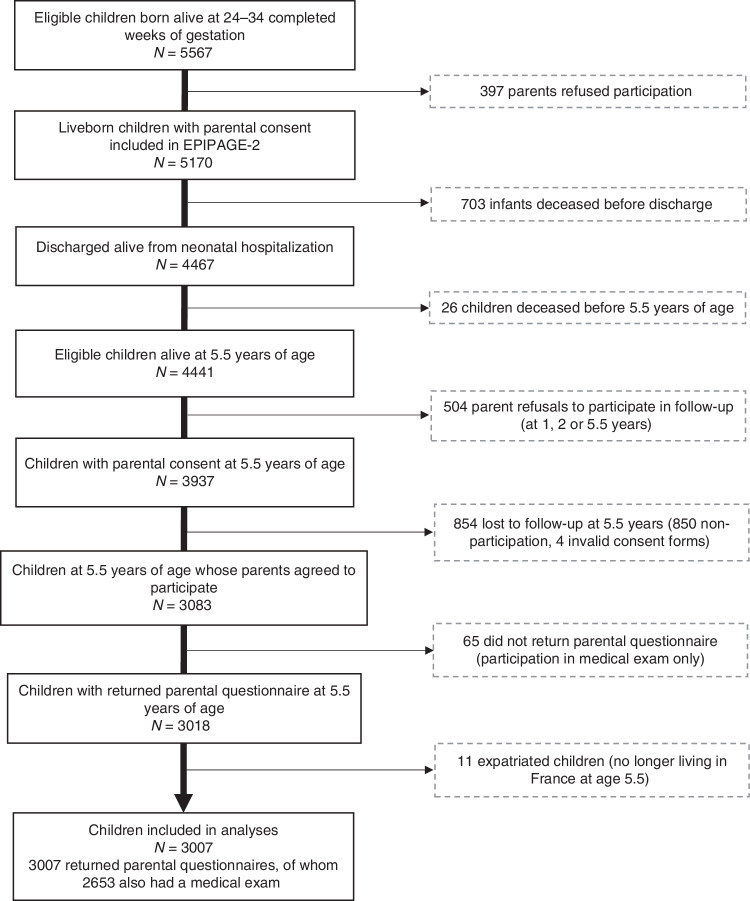
Flow chart of study population.

Out of 5567 children born alive at 24–34 completed weeks’ GA, 4467 were discharged alive from neonatal hospitalization, 4441 were alive at 5 years of age and 3007 participated in the study (67.7% of eligible children) (Fig. 1). The mean age at participation was 5 years and 8 months. Of the participating children, 55.3% were male. The median GA was 32 weeks (IQR 30–34), and the mean BW 1740 g (IQR 1350–2100) (Supplementary Table S2). Over one-third (37.3%) were multiples, and 13.3% had severe neonatal morbidity. Compared to participants, non-participating children were to a larger extent female and singleton, born at later GA and born to multiparous, younger, foreign-born mothers, and to mothers with a low educational level (Supplementary Table S2).

Most children (99.3% overall, 95.1% of children with MSNDI) were enrolled in mainstream school at 5 years of age, of whom most (98.1% and 93.6%, respectively) were in the expected level for their age, and participated full-time (98.6% and 93.0%) (Table 1). Among children in mainstream school, 10.6% had a Personalized Accommodation Plan or Personalized Schooling Plan. Proportions were higher in children born at 24–27 weeks’ GA (20.9%) or with MSNDI (31.5%). School support was received by 8.9% of all children in mainstream school. The most common type of school support was having a school support assistant or participation in a special education classroom.

**Table 1:** School participation and support at 5.5 years of age.

	Gestational age (completed weeks) *n* (weighted %) or *weighted median [IQR]*			Neurodevelopmental impairment *n* (weighted %) or *weighted median [IQR]*			Total *n* (w.%) or *w. median [IQR]*
	24–27	28–31	32–34	Moderate/severe	Mild	None	
	606	1646	755	348	901	1343	
	*N* = 3007			*N* = 2592			*N* = 3007
**School participation**							
*Type of schooling*							**2978**
Mainstream school	593 (98.4)	1610 (99.0)	746 (99.6)	330 (95.1)	**892 (99.9)	1327 (99.9)	2949 (99.3)
Home schooled	1 (0.2)	8 (0.5)	1 (0.1)	3 (1.3)	2 (11.7)	3 (0.1)	10 (0.2)
Specialised institution	8 (1.2)	8 (0.5)	2 (0.3)	11 (3.5)	0 (0.0)	0 (0.0)	18 (0.4)
No schooling	1 (0.1)	0 (0.0)	0 (0.0)	1 (0.1)	0 (0.0)	0 (0.0)	1 (0.01)
*Among children in mainstream schooling:*							
Level of schooling							**2947**
Delayed level for age (first or second year of preschool)	34 (5.7)	**27 (1.7)	7 (0.9)	34 (5.8)	**11 (1.3)	2 (0.2)	68 (1.5)
Usual level for age (third year of preschool)	558 (94.1)	1574 (97.8)	735 (98.7)	293 (93.6)	879 (98.3)	1323 (99.6)	2867 (98.1)
In advance for age (primary school)	1 (0.2)	8 (0.5)	3 (0.4)	3 (0.6)	2 (0.4)	2 (0.2)	12 (0.4)
Participation time							**2914**
Full time	562 (95.6)	**1555 (97.8)	731 (99.3)	290 (93.0)	**875 (99.5)	1310 (99.9)	2848 (98.6)
Part time	26 (4.4)	35 (2.2)	5 (0.7)	34 (7.0)	8 (0.5)	4 (0.1)	66 (1.4)
Age at school entry (chronological age)							**2911**
≤3 years (official age at school start)	557 (94.9)	**1546 (97.4)	723 (98.1)	297 (95.3)	*855 (98.5)	1305 (98.8)	2826 (98.4)
≥4 years (delayed start)	30 (5.1)	41 (2.6)	14 (1.9)	25 (4.8)	18 (1.5)	14 (1.2)	85 (1.6)
**School support (among children in mainstream schooling)**							
Personalised accommodation plan or personalised schooling plan has been established	119 (20.9)	**206 (13.3)	60 (8.2)	118 (31.5)	**106 (9.8)	99 (6.8)	385 (10.6)
School support currently received (any of the below)	117 (20.8)	**174 (11.7)	44 (6.3)	147 (38.6)	**92 (8.4)	31 (2.1)	335 (8.9)
School support assistant^a^ or special education classroom^b^	97 (16.5)	**131 (8.3)	28 (3.8)	129 (32.4)	**62 (5.4)	13 (0.7)	256 (6.1)
Other professional assistance^c^	27 (4.8)	38 (2.5)	21 (2.9)	26 (7.8)	29 (2.9)	14 (1.1)	86 (2.9)
Technical or material aid^d^	9 (1.7)	22 (1.5)	6 (0.8)	21 (5.6)	**8 (0.7)	2 (0.2)	37 (1.1)
Other unspecified aid	12 (2.1)	24 (1.6)	5 (0.7)	18 (3.7)	**10 (1.4)	5 (0.2)	41 (1.0)

*p < 0.01, **p < 0.001;

^a^Auxiliaire de vie scolaire (AVS);

^b^Inclusive classroom or special education classroom (*Unités localisées pour l’inclusion scolaire*, ULIS and *Classe pour l’inclusion scolaire*, CLIS), teaching unit for children with autism, or support from teacher or psychologist from a special needs support network (*Réseau d’aides spécialisées aux élèves en difficulté*, RASED);

^c^Such as extra learning support or support teacher, other than school support assistant (AVS);

^d^For instance, adapted seat or table, inclined reading support, adapted computer or equipment, wheelchair or hearing aid.

School support was more often received by boys (11.0% vs. 6.3% for girls), children born at 24–27 weeks’ GA (20.8% vs. 6.3% for 32–34 weeks’ GA), and children with a BW<3^rd^ centile (14.2% vs. 7.8% for ≥3^rd^ centile), a severe neonatal morbidity (21.5% vs. 7.4% for children without), or a MSNDI (38.6% vs. 8.4% for mild and 2.1% for no impairment) (Table 2). Cerebral palsy, hearing, vision and cognitive impairment, developmental coordination disorder, and behavioural difficulties were each associated with school support in the univariable analyses, with the highest proportion of school support in children with hearing impairment (63.3%). School support was also more commonly received by children with young mothers (15.4%) compared to older mothers (~ 8%) and mothers with a low educational level (12.4%) compared to high (6.5%), and foreign-born (13.0%) compared to native-born mothers (8.2%). The proportion of children receiving school support varied across regions, between 5.7% and 14.5%, representing 7 and 20 children respectively, given the reduced sample size when analysing data by region.

**Table 2:** Any school support by child and family characteristics and region, among children in mainstream school.

	School support *n*/*N* (weighted %) or weighted median [IQR]	*p*-value
**Child and birth-related characteristics**		
Sex		<0.001
Male	211/1453 (11.0)	
Female	124/1288 (6.3)	
Gestational age (completed weeks)	*31 [28*–*33]*	
24–27	117/547 (20.8)	<0.001
28–31	174/1490 (11.7)	
32–34	44/704 (6.3)	
Birthweight		<0.001
<3^rd^ centile	94/580 (14.2)	
3^rd^–9^th^ centile	39/345 (7.8)	
≥10^th^ centile	202/1815 (7.6)	
Multiple birth		0.63
Singleton	230/1778 (9.1)	
Multiples (twins, triplets or quadruplets)	105/963 (8.5)	
Parity		0.75
Primiparous	196/1568 (9.0)	
Multiparous	135/1149 (8.6)	
Severe neonatal morbidity^a^		<0.001
No	192/1984 (7.4)	
Yes	137/642 (21.5)	
**Child’s neurodevelopment at age 5**		
Cerebral palsy^b^		<0.001
No	262/2607 (7.6)	
Yes	71/119 (49.5)	
Hearing impairment^c^		<0.001
None	291/2577 (8.1)	
Mild	17/65 (13.2)	
Moderate or severe	14/23 (63.3)	
Vision impairment^d^		<0.001
None	210/2252 (6.8)	
Mild	24/117 (11.0)	
Moderate or severe	6/13 (39.0)	
*Assessments done in a subpopulation of children who participated in medical and neuropsychological exam at 5 years (N* = *2653)*		
Cognitive impairment^e^		<0.001
None	76/1540 (3.3)	
Mild	67/589 (9.4)	
Moderate or severe	106/234 (34.4)	
Developmental coordination disorder^f^		<0.001
No	79/1859 (3.7)	
Yes	33/154 (15.9)	
Behavioural difficulties^g^		<0.001
No	213/2174 (6.7)	
Yes	67/238 (21.5)	
Neurodevelopmental impairment^h^		<0.001
None	31/1235 (2.1)	
Mild	92/836 (8.4)	
Moderate or severe	147/297 (38.6)	
**Family sociodemographic characteristics**		
Maternal age at delivery	*30 [25*–*34]*	
<25 years	64/368 (15.4)	<0.01
25–34 years	204/1,754 (8.1)	
34< years	67/619 (7.9)	
Mother’s current educational level		<0.001
Low (Upper secondary or lower)	185/1197 (12.4)	
Intermediate (Post-secondary, non-tertiary or short cycle tertiary)	69/693 (5.8)	
High (Bachelor degree or equivalent, or higher)	79/846 (6.5)	
Child’s family situation		0.05
Child lives with single parent	50/367 (12.0)	
Child lives with two parents	263/2277 (8.0)	
Mother’s country of birth		0.01
France	263/2273 (8.2)	
Outside of France	72/459 (13.0)	
**Region**		
Grand Est Alsace	27/214 (11.0)	0.79
Nouvelle Aquitaine	9/138 (8.1)	
Auvergne-Rhône-Alpes	44/363 (8.7)	
Normandie	15/149 (12.4)	
Bourgogne-Franche-Comté	11/101 (6.4)	
Bretagne	13/118 (7.6)	
Centre-Val de Loire	7/62 (5.7)	
Overseas territories	16/141 (7.8)	
Ile-de-France	64/505 (8.0)	
Occitanie	33/252 (9.1)	
Les Hauts de France	51/382 (7.8)	
Provence-Alpes-Côte d’Azur	25/184 (9.5)	
Pays de Loire	20/132 (14.5)	

^a^Severe brain lesion (intraventricular haemorrhage grade III-IV or cystic periventricular leukomalacia), necrotising enterocolitis (Bell’s stages 2–3), severe retinopathy of prematurity (stage >3), severe bronchopulmonary dysplasia (oxygen for at least 28 days and persistent need for oxygen or ventilatory support at 36 weeks’ postmenstrual age) and/or late onset sepsis (positive blood culture after 72 h of life, associated with antibiotic administration for five days or more).

^b^Cerebral palsy: Gross Motor Function Classification System (GMFCS) level 1–5 based on medical examination or postal questionnaire completed by parents.

^c^*None:* No hearing loss, or hearing reported as normal; *mild:* hearing loss ≤40 dB; *moderate or severe:* unilateral or bilateral hearing loss >40 dB not corrected or partially corrected with hearing aid, or deafness.

^d^*None:* binocular visual acuity ≥5/10 or eye consultation within the past 12 months and vision reported as normal; *mild:* binocular visual acuity 3.2–4.9/10; *moderate or severe:* bilateral binocular visual acuity <3.2/10 or <1/10 (blindness), measured with the Sander-Zanlonghi scale (with glasses if needed) or parent-report (postal questionnaire).

^e^*None:* a full scale intelligence quotient (FSIQ) score higher than 1 standard deviation (SD) below the reference mean; *mild:* an FSIQ score between 1 to 2 SD below the reference mean; *moderate or severe:* an FSIQ score lower than 2 SD below the reference mean, evaluated with the Wechsler Preschool and Primary Scale of Intelligence (WPPSI-IV) based on the FSIQ distribution of a reference sample born at 37–40 weeks.

^f^Total Movement Assessment Battery for Children (M-ABC) 2^nd^ edition score ≤5^th^ centile based on the distribution in a reference sample born at 37–40 weeks.

^g^Total Strengths and Difficulties Questionnaire (SDQ) score ≥90th centile, based on a reference sample born at 37–40 weeks.

^h^*None:* no cognitive impairment, cerebral palsy, developmental coordination disorder, hearing or vision impairment, nor behavioural difficulties; *mild:* mild cerebral palsy, visual or hearing impairment, or cognitive impairment, or any behavioural difficulties, and/or developmental coordination disorder, but no moderate or severe impairment; *moderate or severe:* moderate or severe cerebral palsy, visual or hearing impairment and/or cognitive impairment.

Descriptive analyses stratified by severity of neurodevelopmental and cognitive impairments showed that rates of school support varied by perinatal and family-related factors within severity groups (Table 3). For instance, rates differed for children with MSNDI born at 24–27 weeks’ (68.2%) and 32–34 weeks’ GA (23.3%), for children with (64.5%) and without (13.4%) severe neonatal morbidities, and for children with mothers with low (35.3%) and high (45.0%) educational level. Some variation was also observed in children with mild NDI, but rates of school support were significantly lower, overall. Similar but less accentuated variation was seen for the group of children with cognitive impairment.

**Table 3:** Proportion of children with school support by severity of neurodevelopmental and cognitive impairment, and by perinatal and family characteristics.

	Neurodevelopmental impairment^a^			Cognitive impairment^b^		
	None *n* (weighted %)	Mild *n* (weighted %)	Moderate or severe *n* (weighted %)	None *n* (weighted %)	Mild *n* (weighted %)	Moderate or severe *n* (weighted %)
Sex						
Male	23 (2.9)	66 (12.0)	79 (36.8)	49 (3.9)	49 (14.3)	53 (33.4)
Female	8 (1.3)	26 (3.4)	68 (41.3)	27 (2.6)	18 (3.1)	53 (35.8)
Gestational age (completed weeks)						
24–27	9 (4.5)	27 (12.4)	64 (68.2)	25 (9.5)	19 (12.2)	47 (65.9)
28–31	15 (2.2)	53 (12.1)	73 (45.3)	42 (5.0)	37 (12.1)	52 (40.3)
32–34	7 (1.9)	12 (6.2)	10 (23.3)	9 (2.1)	11 (7.9)	7 (20.0)
Birthweight						
<3^rd^ centile	5 (3.0)	21 (7.4)	45 (41.6)	17 (4.5)	10 (5.4)	38 (40.6)
3^rd^–9^th^ centile	9 (4.7)	5 (2.3)	21 (38.9)	12 (4.5)	5 (3.5)	16 (34.9)
≥10^th^ centile	17 (1.4)	66 (9.8)	81 (36.6)	47 (2.8)	52 (11.7)	52 (30.1)
Severe neonatal morbidity^c^						
No	24 (2.2)	57 (7.3)	71 (13.4)	51 (3.1)	41 (8.2)	51 (27.2)
Yes	7 (2.4)	34 (16.2)	72 (64.5)	24 (6.3)	25 (17.8)	52 (63.1)
Maternal age at delivery						
<25 years	4 (5.0)	15 (10.2)	25 (36.4)	12 (6.6)	9 (9.8)	22 (38.0)
25–34 years	22 (2.1)	55 (7.6)	90 (36.7)	49 (3.3)	42 (8.7)	60 (30.2)
34< years	5 (1.2)	22 (9.6)	32 (46.4)	15 (2.2)	16 (11.1)	24 (45.6)
Mother’s current educational level						
Low (Upper secondary or lower)	18 (4.1)	47 (9.0)	80 (35.3)	37 (5.4)	36 (9.7)	62 (31.9)
Intermediate (Post-secondary, non-tertiary or short cycle tertiary)	6 (0.7)	20 (4.5)	33 (43.3)	21 (2.1)	11 (3.7)	25 (40.6)
High (Bachelor degree or equivalent, or higher)	7 (1.4)	24 (9.8)	33 (45.0)	18 (2.3)	19 (13.2)	18 (36.9)
Mother’s country of birth						
France	23 (5.3)	72 (9.3)	116 (33.2)	65 (3.1)	50 (9.6)	79 (35.1)
Outside of France	8 (1.7)	20 (8.3)	31 (40.3)	11 (5.6)	17 (9.1)	27 (32.5)

^a^*None*: no cognitive impairment, cerebral palsy, developmental coordination disorder, hearing or vision impairment, nor behavioural difficulties; *mild*: mild cerebral palsy, visual or hearing impairment, cognitive impairment, or any behavioural difficulties, and/or developmental coordination disorder, but no moderate or severe impairment; *moderate or severe*: moderate or severe cerebral palsy, visual or hearing impairment and/or cognitive impairment.

^b^*None:* a full scale intelligence quotient (FSIQ) score higher than 1 standard deviation (SD) below the reference mean; *mild:* an FSIQ score between  1 to 2 SD below the reference mean; *moderate or severe:* an FSIQ score lower than 2 SD below the reference mean, evaluated with the Wechsler Preschool and Primary Scale of Intelligence (WPPSI-IV) based on the FSIQ distribution of a reference sample born at 37–40 weeks.

^c^Severe brain lesion (intraventricular haemorrhage grade III-IV or cystic periventricular leukomalacia), necrotising enterocolitis (Bell’s stages 2–3), severe retinopathy of prematurity (stage >3), severe bronchopulmonary dysplasia (oxygen for at least 28 days and persistent need for oxygen or ventilatory support at 36 weeks’ postmenstrual age) and/or late onset sepsis (positive blood culture after 72 h of life, associated with antibiotic administration for five days or more).

The aRR of having school support was higher for boys (aRR = 1.37, 95%CI = 1.11–1.68), increased with decreasing GA (aRR = 2.78, 95%CI = 1.84–4.21 at 24–27 weeks’ GA), and was the highest for children with MSNDI (aRR = 17.25, 95%CI = 11.60–25.65, compared to aRR = 4.10, 95%CI = 2.70–6.22 for children with mild NDI) (Table 4). No statistically significant differences were found in school support by sociodemographic characteristics after adjusting for the childrens’ health and development. After adjusting for covariates, the highest aRR of receiving school support was observed in Bourgogne-Franche-Comté (aRR = 1.39, 95%CI = 0.86–2.25) and the lowest in the overseas territories (aRR = 0.53, 95%CI = 0.32–0.87) (Supplementary Fig. S1). No statistically significant differences were seen between regions, with the exception of the overseas territories.

**Table 4:** Relative risk of having school support by perinatal, neurodevelopmental and sociodemographic risk factors.

	Model I^a^		Model II^a^		Model III^a^	
	N. observations: 2740		N. observations: 2368		N. observations: 2356	
	aRR	95% CI	aRR	95% CI	aRR	95% CI
Region: not shown						
Sex: male (ref.: female)	1.60**	[1.29–1.98]	1.37*	[1.11–1.69]	1.37*	[1.11–1.68]
Gestational age (ref.: 32–34 weeks)	**		**		**	
24–27 weeks	3.57**	[2.50–5.10]	2.60**	[1.73–3.90]	2.78**	[1.84–4.21]
28–31 weeks	1.83*	[1.29–2.60]	1.77*	[1.18–2.65]	1.84*	[1.22–2.79]
Birthweight (ref.: ≥10^th^centile)	*					
<3^rd^ centile	1.56*	[1.23–1.97]	1.04	[0.82–1.32]	1.05	[0.83–1.34]
3–9^th^ centile	1.16	[0.84–1.60]	1.14	[0.85–1.54]	1.12	[0.83–1.52]
Neurodevelopmental impairment (ref.: none)^b^			**		**	
Mild			4.13**	[2.75–6.22]	4.10**	[2.70–6.22]
Moderate or severe			17.70**	[12.13–25.81]	17.25**	[11.60–25.65]
Mother’s age at delivery (ref.: 25–34 years)						
<25 years					0.89	[0.67–1.19]
34< years					1.14	[0.89–1.46]
Mother’s educational level (ref.: high)						
Low (Upper secondary or lower)					1.15	[0.88–1.51]
Intermediate (Post-secondary, non-tertiary or short cycle tertiary)					1.05	[0.78–1.41]
Mother’s country of birth: outside of France (ref.: France)					0.98	[0.73–1.30]

*p < 0.01, **p < 0.001;

^a^All models were adjusted for region, and the adjusted relative risk (aRR) for each region, based on model III, are shown in Supplementary Fig. 1. All other covariates for each model are displayed (with aRR and 95% CI) in the table. No additional variables were adjusted for.

^b^*None:* no cognitive impairment, cerebral palsy, developmental coordination disorder, hearing or vision impairment, nor behavioural difficulties; *mild*: mild cerebral palsy, visual or hearing impairment, cognitive impairment, or any behavioural difficulties, and/or developmental coordination disorder, but no moderate or severe impairment; *moderate or severe*: moderate or severe cerebral palsy, visual or hearing impairment and/or cognitive impairment.

## Discussion

This study showed that the vast majority of five-year-old children born before 35 weeks’ GA in France were enrolled in mainstream school at their expected level, even when they had MSNDI. School support, including inclusive and special education classrooms, school support assistants and other types of professional and technical aids at school, was received by 9% of the cohort. Rates of school support was higher among boys, children born at the lowest GA, and those with moderate to severe cognitive or other NDI. However, 61% of children with MSNDI and 92% of children with mild NDI were not receiving school support. The crude proportions of school support varied across regions, but statistically significant lower rates were only found for French overseas territories after adjusting for covariates.

Our results show that the majority of children born before 35 weeks’ GA, including children with MSNDI, were in mainstream school at 5 years of age. Although the most important determinant of receiving school support was the presence of an NDI, not all children with NDI received support. High mainstream school participation is expected, as inclusive schooling principles were formally established in France in 2013,^36^ integrating children with disability or special educational needs when possible. However, around 6% of the children born extremely preterm or with a MSNDI were attending school at a lower-than-expected level, and around 5% had delayed school entry. A delayed school entry could be preferable for some children because of their immaturity^37^ compounded by their earlier birth date due to premature birth; this double disadvantage has been associated with not attaining the expected development at school.^11^ On the other hand, later school entry may delay the provision of special support at a crucial age for development, and the evidence on its benefits shows mixed results.^37^ We did not have data on the reasons for delayed school entry, and further studies are needed to assess the characteristics of children with delayed entry (e.g., if they are born later in the calendar year) and to determine the effects of delayed entry on receiving school support, learning, and academic performance, in these children.

There may be several reasons for the low rate of school support found in our study. It is possible that these children are receiving other types of support outside of school, such as physiotherapy, speech therapy, psychomotor therapy or extra classes, not organized by schools or the Departmental Medical Home for Persons with Disabilities, which may be sufficient, especially if the child has mild impairment. In the case of mild NDI, it is also possible that the need for school support is missed, or takes longer to identify, which could explain the low rates of school support in this subgroup. In addition, obtaining a diagnosis, and the administrative process for obtaining support can be long, meaning that some children may be waiting for requested support. It is possible that some impairments, identified during the clinical examinations in our study, were not observed by the teachers or previously known by the parents, or that all NDI may not directly translate into functional impairments requiring support in the academic setting at 5 years of age. However, previously published results from the EPIPAGE-2 cohort suggests that parents worry about their child’s health and development to a higher extent than health and developmental problems are reported by clinicians.^5^ Parental as well as child perspectives of functional needs are important and should be integrated into future studies on school support decisions, to better understand their subjective experience in relation to support received.

School support was more likely to be received by boys and by children born at the lowest GA after adjusting for NDI, perinatal morbidities, sociodemographic characteristics and region of residence. Previous studies have shown that boys are more likely to receive school support compared to girls,^10,13^ possibly due to more externalizing presentation of difficulties compared to girls,^13,38^ whose needs may be missed or ignored. Children born at later GA may be less included in follow-up networks and programmes for PTB babies, as inclusion is often based on GA (< 32 weeks’ GA), which could explain the decreasing rate of school support with increasing GA; these programmes are designed to detect health, developmental and learning needs early, and may help direct parents towards appropriate support services.^39^

While family socioeconomic and demographic factors were associated with school support in unadjusted analyses, rates of school support were similar after adjusting for perinatal risk factors and NDI. This finding is encouraging, as social factors have been found to act as barriers for accessing services, such as PTB follow-up programmes,^40^ and healthcare services.^41^ It could be that, if difficulties are discovered and support is initiated by school staff, inequities linked to parents’ socioeconomic status have smaller impact on the likelihood of receiving services, especially if the staff is aware of the importance of providing support to children from disadvantaged backgrounds. Higher rates of school support have previously been found among very PTB children with social vulnerabilities^10,31^ after adjusting for perinatal risk factors for neurodevelopment. Given that children with both neurodevelopmental impairment and social vulnerabilities may have additional, sometimes complex needs,^42^ more school support might be desirable in children with social vulnerabilities, to help promote equitable school opportunities and to prevent social inequities. It is also possible that bias linked to loss to follow-up in the cohort, primarily associated with socioeconomic factors, may have masked possible socioeconomic disparities in this study. Further studies are needed to assess possible social inequities in school support provision, and whether the impact of not receiving school support affects children the same way depending on their parents’ educational level; children of parents with higher educational level may have better access to school-related support in the home, and equitable provision of school support, taking into account social vulnerabilities, may be more important than equal access to school support.

Rates of school support varied across regions. However, after adjusting for covariates, a statistically significant difference in school support was only observed for the overseas territories, where children were less likely to receive school support. Geographic disparities in school support could be expected, as financial and professional resources for such support are likely to vary geographically. In addition, schools in more deprived areas may serve populations with particularly high needs for school support, while having less resources to provide it. The five overseas territories, although their contextual settings vary, have overall higher poverty levels and less resources compared to mainland France,^43^ suggesting that there may be geographic inequities in the provision of school support linked to resources, particularly in these regions. Our study lacked data on local resources and deprivation rates, and had insufficient statistical power to explore these geographic differences in more detail.

This study’s strengths are use of a national, population-based cohort of children born before 35 weeks’ GA in all but one French region in 2011. Further, health and neurodevelopmental data were derived from clinical exams using standardized assessment tools, with cut-offs based on a contemporary sample of children born at term, while parents provided data on socioeconomic and demographic characteristics and school support received. The characteristics of our study sample, including the rate of multiple births, are consistent with previous French^44,45^ and European^46^ VPT-cohorts. Nevertheless, several limitations should be considered when interpreting the results. Out of the eligible children, 68% participated with a parental questionnaire, and 60% participated in medical exams at 5 years of age. Because all children had baseline information, we were able to assess the characteristics of children lost to follow-up, primarily associated with mothers being younger, having a lower educational level and being born outside of France, which might have led to an underestimation of the impact of sociodemographic characteristics on receiving school support. Overall, health and developmental problems were not major contributors to attrition in the cohort (Supplementary Table S2). However, previous analyses on the attrition in the EPIPAGE-2 cohort show that children not participating in the five-year follow-up have slightly higher rates of CP assessed at two years corrected age among children with the lowest GA (24–26 weeks) and higher rates of developmental delay assessed at two years corrected age in the group of children born at 27–31 weeks’ GA.^5^ It is therefore possible that we slightly underestimate the rates of school support. Furthermore, the number of children was small in certain subgroups and, in particular, for the regional analyses. Finally, we were unable to assess the impact of available resources for school support, as this data was not available.

## Conclusion

Our results show that inclusive schooling principles are in place in France, and highlight novel results on the role of family sociodemographic characteristics in receiving school support among children born before 35 weeks’ GA in France. We found no significant social disparities in reception of school support, after adjusting for children’s health and development. However, the prevalence of school support was low, below 10% overall, and over 60% of children with MSNDI were not receiving support, raising questions about whether unmet needs for school support exist among children born preterm. Our results call attention to the need for research on support services available in schools, decision-making processes for allocating these resources and qualitative data on parents’ experiences, to understand why some children are not receiving school support despite having MSNDI and cognitive impairments. Research should also explore how the organization of health and educational services affects how inclusive schooling principles are implemented and how the lack of school support affects children’s wellbeing and future academic performance, especially in subgroups of children with social vulnerabilities.

## Supplementary information

Supplementary material Figure S1

Supplementary material Table S1

Supplementary material Table S2

## Data Availability

The data that support the findings of this study are not publicly available. Data from the EPIPAGE-2 study are accessible to any public or private research team, French or foreign, under the conditions ensuring data security and confidentiality specified in the data access charter. Access requires a request to the EPIPAGE-2 Data Access Committee via the cohort webpage: https://epipage2.inserm.fr/index.php/en/related-research/265-data-access-and-questionnaires
