# Supplementary material for: Neurodevelopmental and social determinants of school support received by children born preterm
Source: Pediatr Res. 2025 Aug 4;99(4):1365–74. doi: 10.1038/s41390-025-04287-4 (PMC13102684; doi:10.1038/s41390-025-04287-4)

Supplementary material: Figure S1. Adjusted relative risks of school support by region, compared to the balanced grand mean, derived from adjusted model III in Table 4 (crude rates of school support in parentheses)

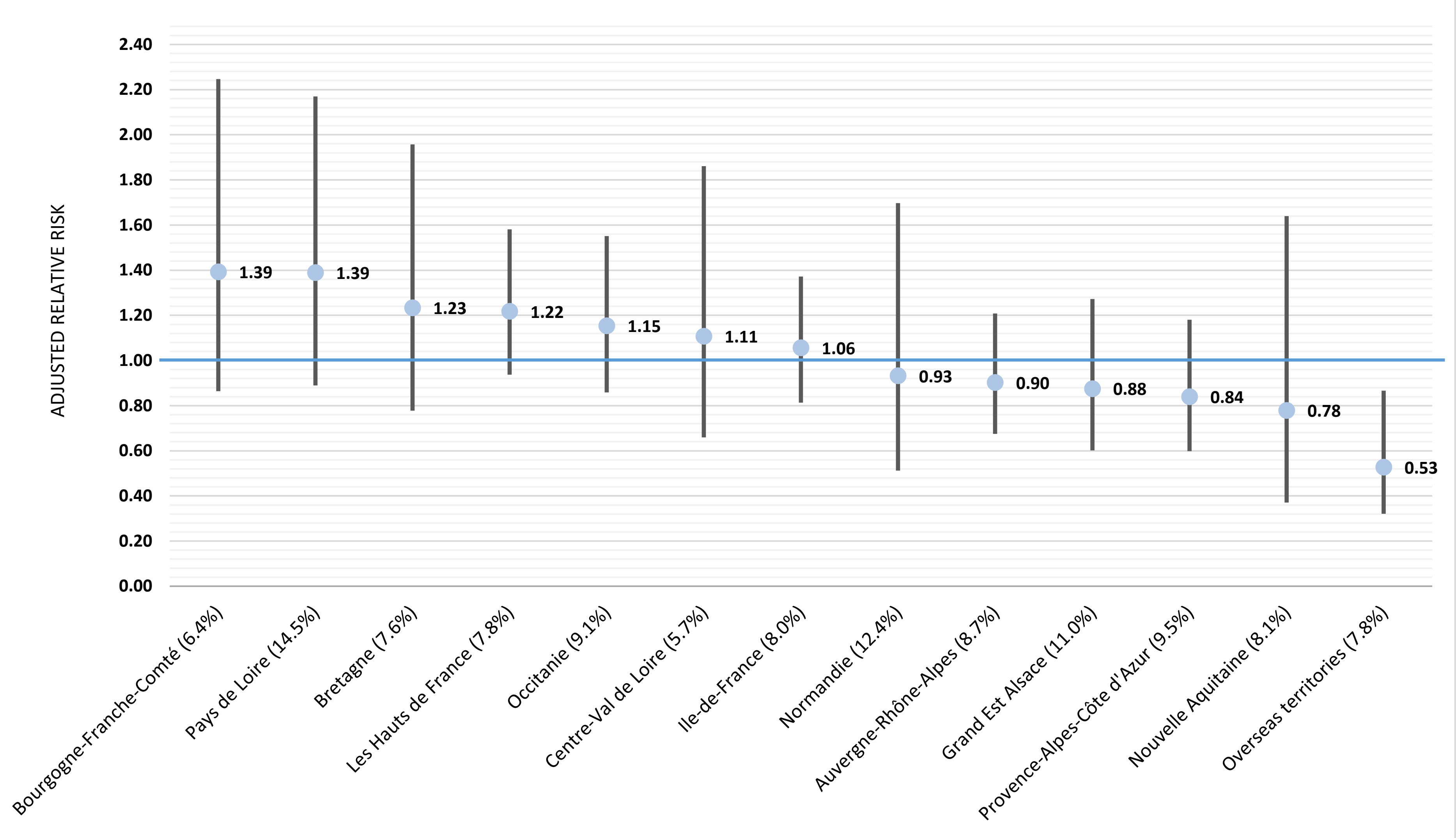

Supplement: Supplementary file 1 — Supplementary material Figure S1 [file 41390_2025_4287_MOESM1_ESM.pdf]
