# Supplementary material for: Neurodevelopmental and social determinants of school support received by children born preterm
Source: Pediatr Res. 2025 Aug 4;99(4):1365–74. doi: 10.1038/s41390-025-04287-4 (PMC13102684; doi:10.1038/s41390-025-04287-4)
Supplement: Supplementary file 2 — Supplementary material Table S1 [file 41390_2025_4287_MOESM2_ESM.pdf]

Supplemental table S1. Translated school-related questions in the EPIPAGE-2 parental questionnaire at 5.5 years of age

Original question in French	English translation
**Votre enfant va-t-il/elle à l'école ?**	**Is your child attending school?**
Oui/non	Yes/No
Si non, où est-il/elle dans la journée ?	If not, where is he/she during the day?
**En quelle classe est-il/elle ?**	**In what class is he/she?**
section des petits en maternelle	First year of preschool
section des moyens en maternelle	Second year of preschool
section des grands en maternelle	Third year of preschool
cours préparatoire de l'école primaire (CP)	First year of primary school
CLIS ou ULIS	Inclusive classroom or special education classroom
établissement spécialisé	Specialized institution
autre, précisez :	Other, precise:
**Va-t-il/elle à l'école :**	**Is he/she going to school:**
toute la journée	Full time
à mi-temps	Part time
de manière irrégulière	Irregularly
**À quel âge il/elle a commencé à aller à l'école?**	**At what age did he/she start attending school?**
Ans, mois	Years, months
**Votre enfant bénéficie-t-il/elle pour sa scolarité :**	**Does your child benefit from any of the following for his/her schooling:**
d'une aide par une auxiliaire de vie scolaire (AVS) ?	School support assistant (AVS)?
d'une aide par un professionnel autre qu'une AVS ?	Other professional aid than AVS?
d'une aide technique (si oui, laquelle ?)	Technical aid (if yes, what?)
d'un Projet d'Accueil Individualisé (P.A.I) ou d'un Projet Personnalisé de Scolarisation (P.P.S)	Personalized Accommodation Plan (P.A.I.) or Personalized Schooling Plan (P.P.S.)
d'un autre type d'aménagement scolaire ?	Other type of schooling aid?
